# Growth‐form and spatiality driving the functional difference of native and alien aquatic plants in Europe

**DOI:** 10.1002/ece3.2703

**Published:** 2017-01-14

**Authors:** Balázs A. Lukács, Anna E. Vojtkó, Attila Mesterházy, Attila Molnár V, Kristóf Süveges, Zsolt Végvári, Guido Brusa, Bruno E. L. Cerabolini

**Affiliations:** ^1^ Department of Tisza ResearchMTA Centre for Ecological Research – DRIDebrecenHungary; ^2^ Hortobágy National Park DirectorateDebrecenHungaryCelldömölkHungary; ^3^ Department of BotanyUniversity of DebrecenDebrecenHungary; ^4^ Department of Conservation ZoologyUniversity of Debrecen‐Hortobágy National Park DirectorateDebrecenHungary; ^5^ Department of Theoretical and Applied Sciences (DiSTA)University of InsubriaVareseItaly

**Keywords:** functional trait, invasion ecology, LDMC, macrophyte, neophyte, SLA

## Abstract

Trait‐based approaches are widely used in community ecology and invasion biology to unravel underlying mechanisms of vegetation dynamics. Although fundamental trade‐offs between specific traits and invasibility are well described among terrestrial plants, little is known about their role and function in aquatic plant species. In this study, we examine the functional differences of aquatic alien and native plants stating that alien and native species differ in selected leaf traits. Our investigation is based on 60 taxa (21 alien and 39 native) collected from 22 freshwater units of Hungarian and Italian lowlands and highlands. Linear mixed models were used to investigate the effects of nativeness on four fundamental traits (leaf area, leaf dry matter content, specific leaf area, and leaf nitrogen content), while the influence of growth‐form, altitude, and site were employed simultaneously. We found significantly higher values of leaf areas and significantly lower values of specific leaf areas for alien species if growth‐form was included in the model as an additional predictor.We showed that the trait‐based approach of autochthony can apply to aquatic environments similar to terrestrial ones, and leaf traits have relevance in explaining aquatic plant ecology whether traits are combined with growth‐forms as a fixed factor. Our results confirm the importance of traits related to competitive ability in the process of aquatic plant invasions. Alien aquatic plants can be characterized as species producing soft leaves faster. We argue that the functional traits of alien aquatic plants are strongly growth‐form dependent. Using the trait‐based approach, we found reliable characteristics of aquatic plants related to species invasions, which might be used, for example, in conservation management.

## 1 Introduction

An increasing number of studies confirmed that alien species serve as dominant forces in ecosystem crises due to their roles as ecosystem engineers by replacing native species and driving local extinctions (Mooney & Cleland, 2001). Thus, researchers have invested large efforts into understanding how species become invasive, why alien species can be more successful than natives, or which environments are most likely to be invaded (Pyšek & Richardson, 2006). All of these questions are related to the invasion paradox (Fridley et al., 2007), providing an explanation for how alien species can be more successful than natives in a more or less natural environment. Native species are generally described as being indigenous to an area since the last Ice Age, whereas aliens have been established due to human activities since then (Pyšek, 1995). Invasive species are those which have been able to overcome a series of geographical, environmental, and dispersal barriers and reproduce successfully in a new environment (Richardson et al., 2000). Both alien and native species are able to become invasive; the latter are also called “expanding natives” (Pyšek, 1995). To date, papers on alien species success mostly focused on terrestrial species; however, aquatic ecosystems are also seriously invaded by alien species (Lukács, Mesterházy, Vidéki, & Király, 2016) and there are papers that aim to study the success and biological attributes of alien aquatic plants (Kliber & Eckert, 2005; Riis et al., 2010).

An increasing number of studies are being published in invasion biology identifying species which are potentially invasive (Pyšek et al., 2012) and attempting to determine which traits enable them to be successful (Fenesi & Botta‐Dukát, 2010; Shea & Chesson, 2002). For this purpose, a trait‐based approach is frequently used, where predominantly whole plant or leaf traits are investigated (e.g., Grotkopp & Rejmanek, 2007; van Kleunen, Weber, & Fischer, 2010; Leishman, Haslehurst, Ares, & Baruch, 2007). Leaf traits are extensively used in plant ecology, being relatively easy to measure and strongly related to plant functions and fitness parameters (Pérez‐Harguindeguy et al. 2013). Green leaves are strongly linked to primary production and carbon accumulation along the “leaf economics spectrum” (LES) (Wright et al., 2004), which describes the trade‐off between the acquisitive and conservative strategies through leaf traits. This trade‐off was found to be globally universal (i.e., independent from growth‐forms and only moderately depending on climate) (Wright et al., 2004).

The applicability of traits in predicting and analyzing biological invasions is nowadays often debated (van Kleunen, Dawson, & Dostal, 2011; Thompson & Davies, 2011), emphasizing that invasive aliens exhibit the same set of traits as successful expanding natives (Leishman, Thomson, & Cooke, 2010). However, other studies revealed that only a handful of traits were universally linked to invasiveness, such as plant height, vegetative spatial growth, specific leaf area (SLA), and other traits related to performance (Pyšek & Richardson, 2007 and literature therein, van Kleunen et al., 2010). In addition, Pyšek and Richardson (2007) concluded that invasiveness is strongly related to leaf traits associated with rapid C capture (high SLA, high leaf area ratio (LAR), and fast relative growth rate), while van Kleunen et al. (2010) found that invasive species have higher trait values for performance‐related traits (reflecting physiology, leaf‐area allocation, shoot allocation, growth rate, size, and fitness).

Hydrophytes are usually neglected from large‐scale comparative trait‐based studies; Poorter, Niinemets, Poorter, Wright, and Villar (2009) provided the only work which applied this group, classifying hydrophytes into a single life‐form category on the one hand and splitting terrestrial species into numerous categories. In this review, the authors found that hydrophytes exhibited the lowest LMA values (i.e., leaf mass area, the reciprocal of SLA, indicating highly acquisitive strategies) compared to a range of terrestrial plant life‐forms. In fact, hydrophytes represent a wide range of life‐history strategies (hereafter “growth‐forms”) (Wiegleb, 1991; Wiegleb et al., 2015). The adaptive strategy of hydrophytes can be directly compared to those of terrestrial species by combining leaf economics and size traits. Besides, their adaptive strategy variation reflects the fundamental trade‐offs in economics that govern all terrestrial plants (Pierce, Brusa, Sartori, & Cerabolini, 2012) so that they could be included in the global spectrum of plant form and function (Díaz et al., 2015). Due to the various economics in contrasting hydrophyte growth‐forms, we suggest that the general models of plant traits comparing alien and native species should also be applied to aquatic species.

Two alternative hypotheses exist to explain the probability of success of alien species: “phenotypic convergence” (Daehler, 2003; Smith & Knapp, 2001) and “phenotypic divergence” (van Kleunen et al., 2010; Lake & Leishman, 2004) depending on whether they found phenotypic similarities or differences between the studied traits in native and alien species. “Phenotypic convergence” is based on the concept of habitat filtering (Weiher, Clarke, & Keddy, 1998), which refers to environmental (abiotic) factors that prevent the establishment or persistence of certain species in a given location; that is, they are “filtered out” based on their traits. This suggests that alien species can only be successful if they are similar to natives. Alternatively, “phenotypic divergence” is related to the concept of limiting similarity (MacArthur & Levins 1967) meaning that competition is strongest between the most similar species. Therefore, by having different traits, alien species can be more successful than natives in the introduced community.

The first aim of the study was to compare alien and native aquatic plant species in terms of four key leaf traits (leaf area, leaf dry matter content, specific leaf area, and leaf nitrogen content) to determine whether we can identify specific traits that might explain the success of alien species over natives. Secondly, we aimed to investigate which hypotheses (“phenotypic convergence” or “phenotypic divergence”) explain the trait composition of co‐occurring native and alien aquatic plant species. Studies aiming at comparing native and invasive plant species by traits usually use key traits that represent independent axes of ecological strategy or niche dimension such as leaf, seed and height traits (Westoby 1998, Ordonez, Wright, & Han, 2010). Height is measured as the difference between the elevation of the highest photosynthetic tissue in the canopy and the base of the plant (Weiher et al., 1999). For hydrophytes, canopy height is difficult to measure where different growth‐forms position leaves equally at water–air interface, but may be free floating or anchored to the substrate. Among seed traits, the average individual seed weight (SWT) is predicted to be the most adequate trait; however, among aquatic plants, vegetative reproduction usually predominates over sexual reproduction (Grace, 1993). In contrast, leaf economics and size traits can reflect adaptive strategy variations among hydrophytes (Pierce et al., 2012).

It is important to note that our alien species are invasive aliens according to the definition of Richardson et al. (2000) (i.e., naturalized plants that produce reproductive offspring, often in very large numbers, at considerable distances from parent plants; <50 years for taxa spreading by seeds and other propagules; >6 m/3 years for taxa spreading by roots, rhizomes, stolons, or creeping stems and thus have the potential to spread over a considerable area). Thus, we conducted invasive–native comparisons. This question focuses only on the ability of species to become invasive and do not consider community invasibility (Pyšek & Richardson, 2006).

## 2 Methods

### 2.1 Database compilation

Trait values of aquatic plant species from 20 water bodies of North Italy were extracted from the TRY database (Kattge et al., 2011). Additional data were obtained from field sampling from two creeks (Hévíz‐creek and Tapolca‐creek) of West Hungary. As a result, our database contains 50 species from Italy and 19 species from Hungary, and nine species were common between the countries. There were three sites contain only alien species and 13 sites have only native species; alien and native species co‐occurred in six sites. At each site, we measured pH, conductivity, water depth, altitude, and latitude as these are the most relevant environmental variables of macrophytes (see Barendregt & Bio, 2003; Lacoul & Freedman, 2006; O'Hare, Gunn, Chapman, Dudley, & Purse, 2012). Among the environmental variables, altitude has been proven to have an effect on trait variation via temperature (Reich, Walters, & Ellsworth, 1997), whereas water depth significantly promotes intraspecific trait variability in macrophytes (Fu et al., 2014). In the sampled watercourses, SECCHI transparency of the water was higher than the average water depth (>2 m); creeks were not shaded by buildings, trees, or shrubs. Thus, we assumed that light availability and photosynthetically active radiation (PAR) were constant and had no effect on plant morphology in any of the sampling sites. In this way, we make sure that leaf traits reflect the spectrum of chemical, structural, and physiological properties of species (Shipley, Lechowicz, Wright, & Reich, 2006). We generally considered it is the best metric to evaluate the differences in species ecological behaviors (Wilson, Thompson, & Hodgson, 1999).

Native species names follow Tutin et al., 2001; and alien species names follow USDA 2016 database, while names of *Nymphaea* cultivars follow Slocum, 2005.

### 2.2 Selection of traits

We followed the same standardized protocol (Cornelissen et al., 2003) as recommended by Pierce et al. (2012): 10 fully expanded, intact leaves of each species were collected from separate individuals. We measured four key traits on all aquatic plant species: (i) Leaf area (LA or leaf size) is strongly related to the energy and water balance of leaves (Cornelissen et al., 2003); (ii) specific leaf area (SLA) is part of the leaf economics spectrum (LES) and strongly correlated with photosynthetic capacity, nitrogen content per leaf mass, and leaf life span (Reich et al., 1999; Wright et al., 2004); (iii) leaf dry matter content (LDMC) reflects the average density of leaf tissues and a trade‐off between the investments in structural tissues versus liquid‐phase processes. Leaf dry matter content is a key variable that governs the correlations among the traits in the leaf economics spectrum (LES), which is considered a robust trait (Roche, Diaz‐Burlinson, & Gachet, 2004) and usually negatively correlated with relative growth rate (Weiher et al., 1999); (iv) leaf nitrogen content (LNC) is calculated as the total amount of nitrogen per unit of dry leaf mass. High values of LNC are associated with high nutritional quality (Cornelissen et al., 2003), which is a predictor of photosynthetic capacity in terms of leaf economics, similar to SLA (Nijs, Behaeghet, & Impens, 1995). This indicates the nitrogen‐use efficiency of plants, which varies with nitrogen availability in the environment, however.

Out of every sampled individual, the youngest, fresh, and healthy but fully developed leaf was collected and scanned using a flatbed desktop scanner and leaf area (LA, mm^2^) was measured using ImageJ (http://imagej.nih.gov/ij) open source image analysis software. The same leaves were weighted in fresh conditions (fresh mass, g) and weighted again after 48 hr of oven‐drying at 80°C (dry mass, mg); then, leaf dry matter content (LDMC, dry mass/fresh mass, mg/g) and specific leaf area (SLA, mm^2^/g = leaf area/dry mass) were calculated. LNC values were measured in three oven dry leaves per species using ICP‐MS (Agilent 8800 triple quad). Individual measurements were averaged for each species.

Species were classified into “*native*” and “*alien*” types based on their native/alien status following the DAISIE 2009 list and Lukács et al., 2016 at the corresponding sampling site. All plants were grouped into growth‐form categories according to Wiegleb (1991) and Wiegleb et al. (2015) (Table 1). We choose Wiegleb's growth‐form system, because the categories are based on leaf morphology which fits best to the aim of the study.

**Table 1:** Hydrophyte growth‐forms according to Wiegleb (1991) and Wiegleb et al. (2015)

Growth‐form	Characteristics	
*Herbid*	Submerged herbaceous plants anchored to sediments; they have usually a terrestrial counterpart	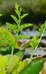
*Myriophyllid*	Anchored submerged plants with long stems and **finely divided submerged leaves**	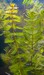
*Nymphaeid*	Anchored plants with **floating entire leaves** attached to a submerged rhizome by an elongate petiole	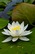
*Peplid*	Anchored plants with elongated or spathulate **leaves forming a terminal rosette adapted for emergence into the atmosphere**	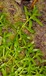
*Pleustophyte*	Plants free floating above the water surface	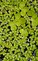
*Potamid*	Anchored plants with **submerged entire** leaves	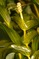
*Vallisnerid*	Anchored plants with **long, floating basal leaves**	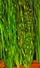

### 2.3 Data analysis

We applied individual trait comparisons to reveal the differences of native and alien species. We used linear mixed models to test whether native and alien species differed significantly in individual traits (LA, SLA, and LDMC). We specified the model in a hierarchical form: The evaluated trait was treated as a response variable; plant type (native or alien), growth‐form, and altitude were treated as a fixed factor, while country, site (nested within country), and species identity were treated as a random factor. The use of site and country as a random factor allowed us to compare native and alien communities co‐occurring under the same environmental conditions, while the use of species identity as a random factor controlled for the possible relatedness of native and alien species. Taxonomic similarity (i.e., congeneric or confamiliar) cannot be considered due to the lack of native‐alien species pairs within genera. To improve normality of predictors, all traits were log_10_‐transformed for all analysis.

During model fitting, we entered and excluded all effects sequentially until only variables explaining significant variation remained. Significance of fixed terms was accepted if *t* > 2.00 (Crawley, 2007). All dropped variables were included again in the model to obtain levels of nonsignificance. We applied the same method to test whether significant effects had not been wrongly excluded. The minimal model was derived by removing terms from the maximal model and adding effects to the simplest model (Pinheiro & Bates, 2000).

All analyses was performed in R environment (R Development Core Team 2009) using the lme4 package (Bates, Mächler, Bolker, & Walker, 2015).

## 3 Results

Environmental variables have low variability in case of latitude (mean = 45.8; min = 45.3; max = 46.8) and water depth (mean = 0.78, min = 0.05; max = 1.6), while pH (mean = 7.5, min = 4.1; max = 10.4) and altitude (mean = 214.06, min = 52; max = 1722) showed great variety.

In total, trait data of 35 native and 18 alien aquatic vascular plant species were collected. Trait means for the 53 species are presented in Table 2. Within native plants, LA ranged from 0.81 mm^2^ in the free floating tiny leaves of *Wolffia arrhiza* to 50,778 mm^2^ in the large entire leaves of *Nymphaea alba*. Within alien species, LA ranged from the free floating tiny leaves of the fern *Azolla caroliniana* (0.92 mm^2^) to the large floating leaves of *Nymphaea rubra* (81,120 mm^2^). Within native species, LDMC values ranged from a low 41.3 mg in *Lemna gibba* to 332.5 mg in the entire floating‐leaved *Potamogeton polygonifolius*. Among alien species, LDMC varied from fine and soft leaves of *Utricularia gibba* (52.2 mg) to herbs such as *Rotala rotundifolia* (374.2 mg). Within native species, SLA values ranged from a moderately low value of 9.70 mm^2^/mg in the large floating‐leaved *Nymphaea alba* to the extremely fine and soft leaves of *Utricularia vulgaris* (163.9 mm^2^/mg). In aliens, SLA values varied from 15.09 mm^2^/mg in herbs such as leaves of *Ceratopteris thalictroides* to 203.2 mm^2^/mg in the fine and soft leaves of *Myriophyllum aquaticum*. Within natives, LNC values ranged from a low 19.1 mg/g in *Nymphaea alba* to 178.9 mg/g of *Nymphaea alba*. Among alien species, LDMC varied from the tiny *Lemna minuta* (26.7 mg/g) to *Nymphaea “bluebird”* (178.9 mg/g).

**Table 2:** Leaf traits and growth‐forms of 60 hydrophyte species

Species	Growth‐form	Nativeness	LA (mm^2^)	LDMC (mg)	SLA (mm^2^/mg)	LNC (mg/g)
*Callitriche obtusangula*	Pepliden	Native	26.76 ± 0.77	79.77 ± 1.72	94.26 ± 1.2	46.7 ± 1.12
*Callitriche platycarpa*	Pepliden	Native	32.03 ± 1.47	68.47 ± 2.74	127.93 ± 3.77	28.07 ± 0.03
*Ceratophyllum demersum*	Pleustophyte	Native	108.68 ± 20.9	46.22 ± 4.41	126.25 ± 14.09	66.89 ± 11.09
*Groenlandia densa*	Potamiden	Native	39.87 ± 2.55	112.14 ± 2.92	173.28 ± 4.79	31.2 ± 0.42
*Hippuris vulgaris*	Myriophylliden	Native	52.28 ± 2.56	73.69 ± 1.85	125.02 ± 2.78	33.67 ± 0.18
*Hottonia palustris*	Pleustophyte	Native	257.74 ± 3.61	45.88 ± 2.73	187.5 ± 11.82	19.83 ± 0.34
*Hydrocharis morsus*‐*ranae*	Pleustophyte	Native	1365.46 ± 57.91	32.41 ± 1.42	151.97 ± 2.86	91.48 ± 22.75
*Lemna gibba*	Pleustophyte	Native	18.79 ± 0.66	56.87 ± 2.17	41.31 ± 3.03	36.7 ± 0.2
*Lemna minor*	Pleustophyte	Native	7.88 ± 0.71	70.05 ± 6.75	267.66 ± 75.7	27.9 ± 0.06
*Lemna trisulca*	Pleustophyte	Native	48.63 ± 16.95	42.57 ± 4.14	151.12 ± 11.26	79.24 ± 23.14
*Marsilea quadrifolia*	Nymphaeiden	Native	534.21 ± 39.19	33.47 ± 0.55	22.75 ± 0.4	31.55 ± 0.3
*Myriophyllum spicatum*	Myriophylliden	Native	111.5 ± 6.21	64.99 ± 2.26	111.28 ± 2.85	36.97 ± 0.15
*Myriophyllum verticillatum*	Myriophylliden	Native	278.35 ± 18.41	96.55 ± 3.97	76.41 ± 1.49	27.17 ± 0.12
*Najas marina*	Potamiden	Native	94.34 ± 5.99	39.83 ± 1.62	48.3 ± 0.96	23.67 ± 0.38
*Najas minor*	Potamiden	Native	6.23 ± 0.41	76.33 ± 5.13	121.41 ± 5.1	36.53 ± 0.44
*Nasturtium officinale*	Herbiden	Native	339.29 ± 50.75	101.01 ± 4.1	6.16 ± 0.16	67.17 ± 0.44
*Nuphar luteum*	Nymphaeiden	Native	27701.7 ± 1559.05	10.42 ± 0.31	20 ± 0.48	27.32 ± 0.15
*Nymphaea alba*	Nymphaeiden	Native	50778.54 ± 3346.24	9.71 ± 0.46	221.89 ± 7.61	95.3 ± 33.97
*Nymphoides peltata*	Nymphaeiden	Native	6894.26 ± 639.2	26.12 ± 1.01	119.15 ± 2.48	27.93 ± 0.03
*Potamogeton berchtoldii*	Potamiden	Native	60.49 ± 2.18	98.33 ± 4.71	178.77 ± 8.13	34.77 ± 0.44
*Potamogeton crispus*	Potamiden	Native	499.91 ± 12.37	45.33 ± 1.52	198.67 ± 5.95	42.13 ± 0.12
*Potamogeton lucens*	Potamiden	Native	1686.2 ± 69.82	41.26 ± 0.6	124.18 ± 1.43	46.7 ± 0.32
*Potamogeton natans*	Nymphaeiden	Native	3736.92 ± 238.7	31.7 ± 1.6	186.1 ± 4.8	40.93 ± 0.55
*Potamogeton nodosus*	Nymphaeiden	Native	4068.4 ± 222.16	24.23 ± 2.62	195.57 ± 15.24	34.9 ± 0.36
*Potamogeton pectinatus*	Potamiden	Native	61.31 ± 10.8	29.79 ± 2.07	170.69 ± 17.1	81.06 ± 19.93
*Potamogeton perfoliatus*	Potamiden	Native	654.39 ± 43.33	40.23 ± 0.94	163.93 ± 3.12	24.5 ± 0.36
*Potamogeton polygonifolius*	Nymphaeiden	Native	1529.03 ± 72.52	14.97 ± 0.34	332.58 ± 5.62	23.4 ± 0.21
*Potamogeton trichoides*	Potamiden	Native	24.36 ± 1.58	80.2 ± 1.8	220.59 ± 5.55	46.47 ± 0.62
*Ranunculus aquatilis*	Myriophylliden	Native	169.52 ± 9.91	42.36 ± 0.7	106.73 ± 1.05	52.93 ± 0.24
*Ranunculus fluitans*	Myriophylliden	Native	638.83 ± 37.34	25.23 ± 1.12	132.28 ± 3.95	31 ± 0.1
*Ranunculus trichophyllus*	Myriophylliden	Native	540.66 ± 103.55	42.52 ± 1.89	147.62 ± 15.47	29.97 ± 0.43
*Salvinia natans*	Pleustophyte	Native	126.48 ± 5.69	56.7 ± 2.77	72.22 ± 2.25	30.8 ± 0.21
*Sparganium emersum*	Vallisneriden	Native	5247.5 ± 555.75	42.5 ± 1.14	96.01 ± 3.12	36.83 ± 0.15
*Sparganium minimum*	Vallisneriden	Native	3042.35 ± 95.67	21.56 ± 0.55	21.01 ± 0.45	36.9 ± 0.12
*Spirodela polyrrhiza*	Pleustophyte	Native	45.86 ± 5.2	42.84 ± 3.51	145.83 ± 38.2	46.98 ± 0.32
*Trapa natans*	Nymphaeiden	Native	3640.73 ± 147.69	11.44 ± 0.2	223.25 ± 3.69	27.8 ± 0.06
*Utricularia australis*	Pleustophyte	Native	106.5 ± 3.95	133.3 ± 6.19	79.99 ± 1.15	40.43 ± 0.39
*Utricularia vulgaris*	Pleustophyte	Native	46.27 ± 3.67	163.98 ± 2.94	80.86 ± 1.88	34.57 ± 0.62
*Wolffia arrhiza*	Pleustophyte	Native	0.81 ± 0.03	103.44 ± 8.16	43.82 ± 2.31	43.1 ± 0.45
*Azolla filiculoides*	Pleustophyte	Alien	0.92 ± 0.05	41.38 ± 3.72	295.08 ± 22.97	35.37 ± 0.19
*Bacopa crenata*	Pepliden	Alien	75.58 ± 7.97	23.03 ± 1.59	148.71 ± 14.54	151.37 ± 0
*Cabomba caroliniana*	Myriophylliden	Alien	1507.67 ± 139.75	108.06 ± 10.12	117.53 ± 14.47	128.66 ± 0
*Ceratopteris thalictroides*	Herbiden	Alien	5477.76 ± 2033.04	15.1 ± 1.27	164.3 ± 11.5	133.32 ± 0
*Elodea canadensis*	Potamiden	Alien	26.26 ± 1.17	76.38 ± 4.52	175.26 ± 9.23	45.17 ± 0.41
*Elodea densa*	Potamiden	Alien	103.98 ± 4.4	92.58 ± 2.19	14.14 ± 0.2	50.63 ± 0.28
*Elodea nuttallii*	Potamiden	Alien	27.73 ± 1.35	62.29 ± 2.56	224.81 ± 4.6	33.23 ± 0.6
*Hydrilla verticillata*	Potamiden	Alien	112.6 ± 16.17	43.94 ± 5.42	191.46 ± 25.09	100.36 ± 0
*Lagarosiphon major*	Potamiden	Alien	17.56 ± 0.85	46.24 ± 0.71	243.62 ± 2.66	29.93 ± 0.24
*Lemna minuta*	Pleustophyte	Alien	3.07 ± 0.58	132.61 ± 14.84	123.09 ± 8.44	26.87 ± 0.09
*Myriophyllum aquaticum*	Myriophylliden	Alien	455.14 ± 18.26	203.2 ± 3.64	68.31 ± 1.06	30.43 ± 0.12
*Nymphaea odorata*	Nymphaeiden	Alien	25388.1 ± 1584.66	12.53 ± 0.59	18.68 ± 0.67	27.51 ± 0.19
*Nymphaea rubra*	Nymphaeiden	Alien	81120.32 ± 21675.02	15.7 ± 3.39	141.3 ± 34.27	NA
*Nymphaea* x *“bluebird”*	Nymphaeiden	Alien	57158.64 ± 4984.57	17.49 ± 0.7	175.98 ± 32.57	178.97 ± 0
*Nymphaea* x *marliacea*	Nymphaeiden	Alien	43936.7 ± 2703.14	13.87 ± 0.7	17.45 ± 1.04	23.88 ± 0.14
*Nymphaea* x “*purpurea”*	Nymphaeiden	Alien	59895.84 ± 3672.88	16.18 ± 1.06	213.82 ± 23.25	162.38 ± 0
*Rotala rotundifolia*	Pepliden	Alien	79.64 ± 6.41	16.73 ± 1.6	374.22 ± 23.31	66.44 ± 0
*Utricularia gibba*	Pleustophyte	Alien	328 ± 90.96	70.37 ± 19.21	52.25 ± 0	NA
*Vallisneria americana*	Vallisneriden	Alien	21861.6 ± 1451.61	43.94 ± 1.47	55.98 ± 1.9	28.5 ± 0.12
*Vallisneria gigantea*	Vallisneriden	Alien	17444.61 ± 2275.25	34.49 ± 3.13	72.26 ± 8.77	119.09 ± 0.06
*Vallisneria spiralis*	Vallisneriden	Alien	3365.66 ± 393.27	56.47 ± 4.82	135.21 ± 33.29	94.69 ± 26.78

Data represent the means ± *SE* of ten (LA, SLA, LDMC) and three (LNC) replicates. Traits are LA, leaf area; LDMC, leaf dry matter content; SLA, specific leaf area; LNC, leaf nitrogen content. Growth‐form follows Wiegleb (1991).

**Figure 1:**
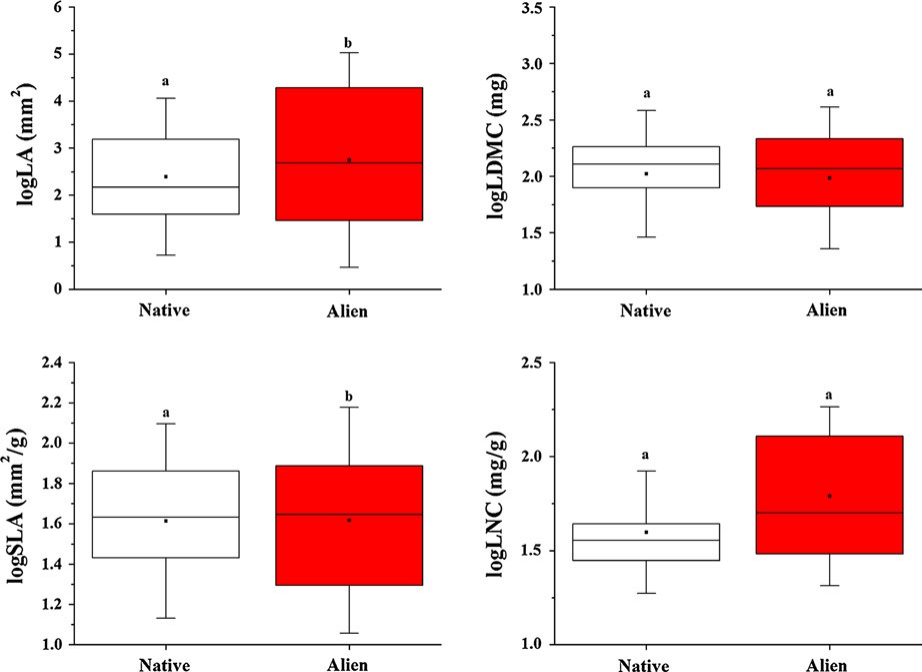
Boxplot of leaf area (LA), leaf dry matter content (LDMC), specific leaf area (SLA), and leaf nitrogen content (LNC) of hydrophyte alien and native species. Values are log‐transformed. Whiskers are standard deviations. Different letters means significant differences between alien (red box) and native (open box) species obtained by linear mixed model, where traits and nativeness was incorporated with growth‐form, environmental variables, spatiality and species identity into one model

**Figure 2:**
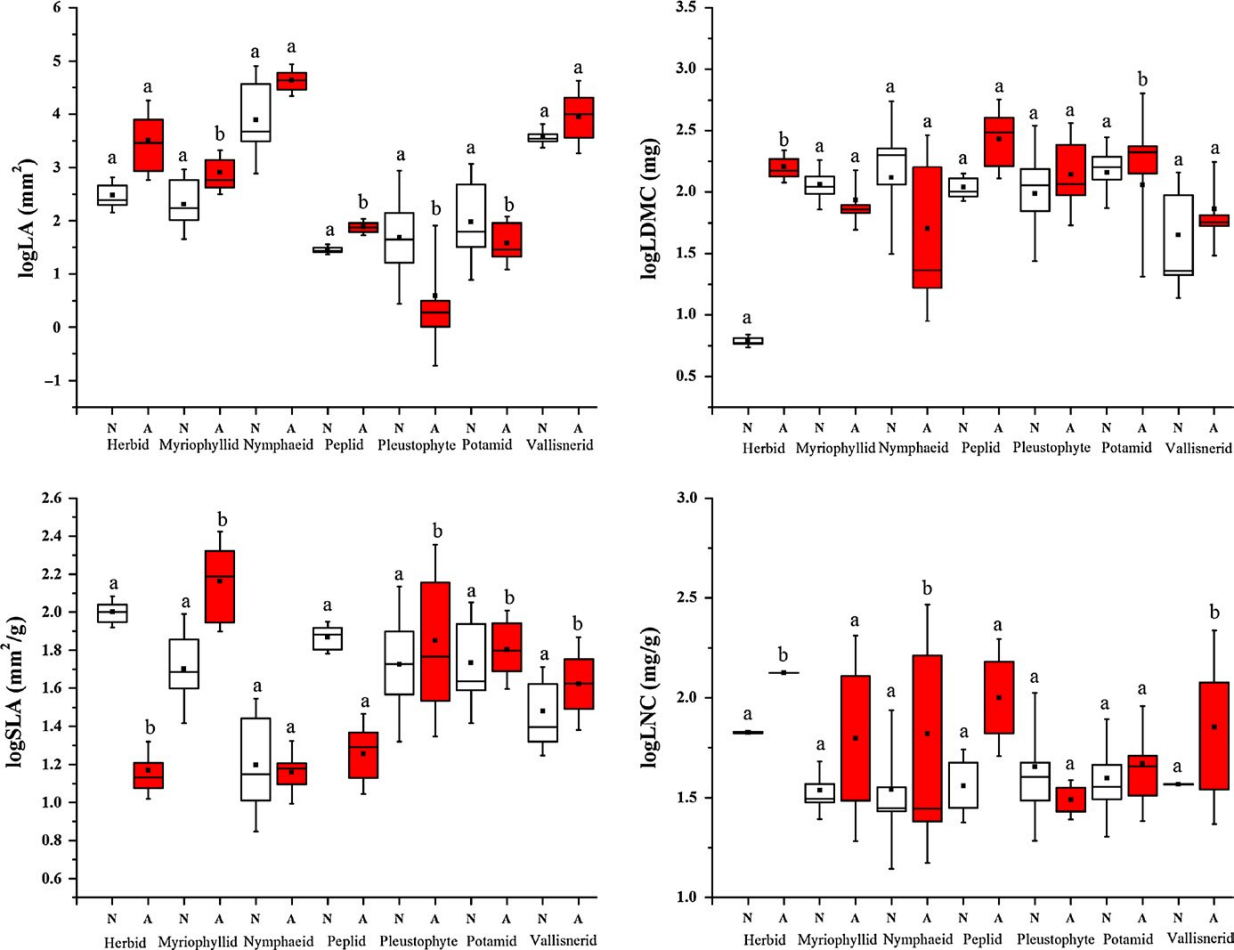
Boxplot of leaf area (LA), leaf dry matter content (LDMC), specific leaf area (SLA) and leaf nitrogen content (LNC) of hydrophyte growth‐forms. Alien and native species plotted separately. Values are log‐transformed. Notations: N‐native; A‐alien. Whiskers are standard deviations. Different letters mean significant differences between alien (red box) and native (open box) species obtained by linear mixed model, where traits and nativeness were incorporated with growth‐form, environmental variables, spatiality, and species identity into one model

Linear mixed model comparisons of individual traits revealed that alien aquatic plant leaves have a substantially higher LA and SLA values than co‐occurring native species when averaged across all species and growth‐forms (Figure 1 and Table 3). Species nativeness was solely important in explaining differences in none of the traits. Nativeness together with growth‐form and water depth was responsible for higher LA; nativeness together with growth‐form and latitude co‐specified the lower SLA. Lower LDMC values of alien aquatic plants were determined by pH and water depth, while higher LNC values of alien species were determined by growth‐form, altitude, and latitude. However, if we consider growth‐forms as individual units, variation of the four leaf traits showed large differences between aliens and natives within each growth‐form (Figure 2).

**Table 3:** Differences of leaf traits between alien and native aquatic vascular plants using final linear mixed models

Trait	Estimate	*SE*	*t*‐value
All			
LA			
*Intercept*	3.468	1.804	1.923
Nativeness	2.894	1.029	2.811*
Growth‐form (*Nymphaeid*) max	2.877	0.823	3.497*
Altitude	0.265	0.884	0.300 NS
Latitude	−1.788	2.973	−0.601 NS
pH	−3.424	1.949	−1.757 NS
Depth	−0.995	0.389	−2.557*
LDMC			
*Intercept*	0.056	0.753	0.075
Nativeness	0.639	0.551	1.159 NS
Growth‐form (*Potamid*) max	0.836	0.423	1.976 NS
Altitude	0.102	0.252	0.406 NS
Latitude	−0.214	0.722	−0.296 NS
pH	2.413	0.855	2.820*
Depth	0.706	0.167	4.229*
SLA			
*Intercept*	36.840	11.591	3.178
Nativeness	−0.695	0.279	−2.494*
Growth‐form (*Nymphaeid*) max	−0.738	0.209	−3.527*
Altitude	0.144	0.095	1.522 NS
Latitude	−20.984	6.978	−3.007*
pH	0.025	0.406	0.061 NS
Depth	−0.059	0.104	−0.565 NS
LNC			
*Intercept*	137.395	28.042	4.900
Nativeness	0.138	0.553	0.249 NS
Growth‐form (*Pleustophyte*) max	−0.294	0.119	−2.478*
Altitude	0.452	0.112	4.024*
Latitude	−94.228	13.766	−6.845*
pH	−0.342	0.378	−0.905 NS
Depth	0.005	0.082	0.059 NS
Herbid			
LA			
*Intercept*	2.481	0.563	4.408
Nativeness	1.029	0.798	1.289 NS
Altitude	Data deficient		
Latitude	Data deficient		
pH	Data deficient		
Depth	Data deficient		
LDMC			
*Intercept*	0.789	0.102	7.751
Nativeness	1.420	0.144	9.840*
Altitude	Data deficient		
Latitude	Data deficient		
pH	Data deficient		
Depth	Data deficient		
SLA			
*Intercept*	2.001	0.155	12.880
Nativeness	−0.832	0.220	−3.782*
Altitude	Data deficient		
Latitude	Data deficient		
pH	Data deficient		
Depth	Data deficient		
LNC			
*Intercept*	1.827	0.006	285.170
Nativeness	0.298	0.009	32.860*
Altitude	Data deficient		
Latitude	Data deficient		
pH	Data deficient		
Depth	Data deficient		
Myriophyllid			
LA			
*Intercept*	0.781	4.251	0.184
Nativeness	−2.466	1.215	−2.029*
Altitude	−0.271	1.043	−0.260 NS
Latitude	−3.672	1.989	−1.846 NS
pH	8.874	8.905	0.996 NS
Depth	−0.666	0.604	−1.102 NS
LDMC			
*Intercept*	6.470	0.743	8.704
Nativeness	−0.095	0.069	−1.385 NS
Altitude	0.814	0.155	5.258*
Latitude	1.328	0.384	3.461*
pH	−9.223	1.335	−6.910*
Depth	−0.077	0.106	−0.721 NS
SLA			
*Intercept*	−2.994	3.795	−0.789
Nativeness	1.134	0.334	3.391*
Altitude	−0.871	0.686	−1.270 NS
Latitude	−0.596	2.204	−0.270 NS
pH	8.363	5.857	1.428 NS
Depth	0.168	0.398	0.424 NS
LNC			
*Intercept*	1.642	4.157	0.395
Nativeness	−0.042	0.202	−0.208 NS
Altitude	−0.329	0.743	−0.443 NS
Latitude	−1.685	1.327	−1.270 NS
pH	3.809	8.154	0.467 NS
Depth	0.341	0.387	0.881 NS
Nymphaeid			
LA			
*Intercept*	3.340	5.780	0.578
Nativeness	0.468	0.276	1.696 NS
Altitude	1.493	1.042	1.433 NS
Latitude	−2.878	3.277	−0.878 NS
pH	1.903	2.747	0.693 NS
Depth	−0.559	0.435	−1.285 NS
LDMC			
*Intercept*	5.409	11.959	0.452
Nativeness	−0.249	0.265	−0.940 NS
Altitude	−0.579	0.680	−0.851 NS
Latitude	0.482	8.244	0.058 NS
pH	−3.227	1.799	−1.794 NS
Depth	0.480	0.328	1.462 NS
SLA			
*Intercept*	1.191	0.076	15.720
Nativeness	0.154	0.116	1.326 NS
Altitude	−0.628	0.372	−1.687 NS
Latitude	−66.962	36.132	−1.853 NS
pH	−0.784	0.733	−1.070 NS
Depth	−0.118	0.148	−0.800 NS
LNC			
*Intercept*	4.556	0.725	6.282
Nativeness	0.197	0.095	2.074*
Altitude	−0.628	0.316	−1.990 NS
Latitude	−0.616	0.436	−1.411 NS
pH	−0.762	0.591	−1.288 NS
Depth	0.221	0.245	0.904 NS
Peplid			
LA			
*Intercept*	7.853	3.000	2.618
Nativeness	−2.786	1.283	−2.171*
Altitude	Data deficient		
Latitude	Data deficient		
pH	−7.139	3.350	−2.131*
Depth	Data deficient		
LDMC			
*Intercept*	−4.184	12.914	−0.324
Nativeness	−0.198	0.855	−0.232 NS
Altitude	Data deficient		
Latitude	Data deficient		
pH	6.758	14.427	0.468 NS
Depth	Data deficient		
SLA			
*Intercept*	13.200	33.871	0.390
Nativeness	−0.390	2.218	−0.176 NS
Altitude	Data deficient		
Latitude	Data deficient		
pH	−12.470	37.839	−0.330 NS
Depth	Data deficient		
LNC			
*Intercept*	−17.261	30.474	−0.566
Nativeness	1.652	1.977	0.836 NS
Altitude	Data deficient		
Latitude	Data deficient		
pH	21.025	34.044	0.618 NS
Depth	Data deficient		
Pleustophyte			
LA			
*Intercept*	466.955	79.017	5.910
Nativeness	−2.805	1.109	−2.530*
Altitude	0.894	0.994	0.900 NS
Latitude	−316.507	46.668	−6.782*
pH	−8.610	1.415	−6.085*
Depth	−1.018	0.436	−2.335*
LDMC			
*Intercept*	−3.170	5.349	−0.593
Nativeness	0.078	0.196	0.401 NS
Altitude	1.266	0.936	1.352 NS
Latitude	0.105	2.119	0.049 NS
pH	2.836	2.549	1.112 NS
Depth	0.839	0.687	1.221 NS
SLA			
*Intercept*	21.369	36.906	0.579
Nativeness	0.728	0.316	2.301*
Altitude	−0.070	0.492	−0.143 NS
Latitude	−12.964	25.644	−0.506 NS
pH	−0.876	1.121	−0.781 NS
Depth	0.295	0.261	1.128 NS
LNC			
Intercept	210.851	86.055	2.450
Nativeness	0.342	0.207	1.655 NS
Altitude	0.390	1.015	0.384 NS
Latitude	−144.133	58.364	−2.470*
pH	−1.693	1.622	−1.043 NS
Depth	−0.175	0.363	−0.482 NS
Potamid			
LA			
*Intercept*	12.215	5.896	2.072
Nativeness	−2.802	1.066	−2.627*
Altitude	−0.776	0.534	−1.452 NS
Latitude	0.859	2.081	0.413 NS
pH	−10.632	6.397	−1.662 NS
Depth	0.638	0.399	1.600 NS
LDMC			
*Intercept*	5.075	2.938	1.727
Nativeness	0.119	0.059	2.019*
Altitude	0.232	0.416	0.558 NS
Latitude	−0.321	1.711	−0.187 NS
pH	−3.369	2.270	−1.484 NS
Depth	0.002	0.074	0.022 NS
SLA			
*Intercept*	32.675	28.130	1.162
Nativeness	0.203	0.073	2.793*
Altitude	0.349	0.153	2.286*
Latitude	−19.199	17.052	−1.126 NS
pH	−1.316	1.347	−0.976 NS
Depth	−0.328	0.091	−3.596*
LNC			
*Intercept*	112.685	58.465	1.927
Nativeness	−0.002	0.098	−0.022 NS
Altitude	0.358	0.264	1.353 NS
Latitude	−76.383	39.689	−1.925 NS
pH	−1.290	2.367	−0.545 NS
Depth	0.020	0.121	0.167 NS
Vallisnerid			
LA			
*Intercept*	3.179	5.214	0.610
Nativeness	0.329	0.585	0.563 NS
Altitude	0.064	0.790	0.081 NS
Latitude	1.518	0.780	1.946 NS
pH	−2.011	5.969	−0.337 NS
Depth	0.836	0.776	1.078 NS
LDMC			
*Intercept*	−118.865	83.718	−1.420
Nativeness	−0.449	0.374	−1.202 NS
Altitude	−1.399	1.126	−1.242 NS
Latitude	71.980	49.126	1.465 NS
pH	4.722	9.437	0.500 NS
Depth	−0.429	1.122	−0.382 NS
SLA			
*Intercept*	3.584	1.421	2.521
Nativeness	0.900	0.344	2.618*
Altitude	0.131	0.215	0.610 NS
Latitude	0.561	0.374	1.498 NS
pH	−3.584	1.934	−1.853 NS
Depth	−0.090	0.225	−0.401 NS
LNC			
*Intercept*	0.679	0.914	0.743
Nativeness	0.609	0.286	2.131*
Altitude	0.291	0.357	0.816 NS
Latitude	0.875	1.094	0.799 NS
pH	−21.716	11.120	−1.953 NS
Depth	Data deficient		

Country, site (nested within country), and species identity were treated as a random factor. Peplid and Vallisnerid growth‐form was omitted from the analyses due to the lack of alien‐native species pairs. Traits with |*t*| > 2.00 mean substantial differences and indicated with an asterisk (*). Traits with |*t*| < 2.00 means non‐substantial differences and indicated with NS

Within growth‐forms, linear mixed model comparison of traits revealed substantial differences between leaf trait values of the corresponding natives and aliens (Figure 2 and Table 3). Alien species exhibited higher LA values within Myriophyllid, Peplid (just as across all species), Pleustophyte, and Potamid species (opposite to all species), whereas no differences were seen within Herbids, Nymphaeids, and Vallisnerids. Substantial differences between leaf areas can also be attributed to pH within Peplids, whereas it can be attributed to latitude, pH, and water depth within Pleustophytes.

LDMC of alien and native species substantially differed among Herbids and Potamids with shifts to the opposite direction which is seen in the all‐species comparison. Differences between LDMC can also be attributed to altitude, latitude, and pH within Myriophyllids.

Alien Myriophyllid, Pleustophyte, Potamid, and Vallisnerid species have substantially higher SLA values (just as across all species), while alien Herbids have substantially smaller SLA. Nativeness has no effects on SLA within Nymphaeid and Peplid species. Differences between SLA can be attributed to altitude and water depth within Potamids.

For LNC values, the group difference was found to be substantial between alien and natives in Herbid, Nymphaeid, and Vallisnerid species (just as across all species). Latitude can be attributed to the substantial differences of LNC within Pleustophytes.

## 4 Discussion

This study is the first comparison of native and alien aquatic plants based on continuous traits. However, a number of studies (Schultz & Dibble, 2012; Thiébaut, 2007) used ordinal trait attributes to analyze alien aquatic plant strategies; none of them applied or measured functional traits on continuous scales. After comparing our study species in terms of their key functional traits, we can conclude that alien and native aquatic plant species differs only in their LA and SLA when all growth‐forms were pooled together (Figure 1). By answering our second question, our results indicate that both the concept of “phenotypic convergence” and “phenotypic divergence” can be applied to alien aquatic plant species depending on which trait we measure. We found convergence in the case of LDMC and LNC which suggest a filtering mechanism for these traits, whereas in the case of LA and SLA, we found divergence; therefore, limiting similarity was the dominant mechanism for these leaf traits. This implies that there is a strong environmental filtering toward a certain amount of solid tissue and nutrients in the leaves to remain compact, whereas at the same time, aliens tend to produce larger leaves and have faster growth rates (via high SLA) and therefore are able to outcompete co‐existing natives.

However, the comparisons of individual traits revealed that growth‐form also have a substantial effect on traits variation (LA, SLA, and LNC). Hydrophytes incorporate several morphologically distinct growth‐forms; thus, species (via growth‐forms) represent different plant strategies as well. We assumed that the large differences between growth‐forms might mask differences between alien and native species; therefore, in order to control for this effect, we compared native and alien species within growth‐forms and obtained significant differences in the case of all four traits (Figure 2). This finding is in line with studies demonstrating that the response of different growth‐form of aquatic plants to local environmental variables (Akasaka & Takamura, 2011; Alahuhta et al., 2013) and the response of plant invasiveness (Hamilton et al., 2005) varied significantly.

LDMC is a key trait of the leaf economics spectrum representing the average density of leaf tissues, positively correlated with leaf life span and negatively correlated with relative growth rate and SLA (Cornelissen et al., 2003). Within growth‐forms, linear mixed model comparison of LDMC values revealed that alien Potamid (*Elodea canadensis, Hydrilla verticillata, Lagarosiphon major*) and alien Herbid (*Ceratopteris thalictroides*) species have less dense tissues than native Potamids (*Groenlandia densa* and *Najas marina*) and native Herbids (*Nasturtium officinale*). Our results are in line with the results of Riis et al. (2012), who found that light availability had an overall strong effect on growth rate and plant morphology if we consider that less dense leaf tissues of alien species might be an advantage in the competition for light. In fact, lower average density of leaf tissues enables aliens to build up their photosynthetic organs faster and more easily and invest less into structural tissue elements. Overall, these taxa can reach faster growth rates (Weiher et al., 1999), which is certainly a competitive advantage.

Our results indicate that leaf area is an important trait in the separation of alien and native aquatic plant species, as it was also proved in the case of terrestrial species (Daehler, 2003; Pyšek & Richardson, 2007). Interspecific variation in LA (and leaf area index—LAI) has been usually related to climatic, geological, altitudinal, and latitudinal factors. Within climatic zones, LA may be linked to ecological strategies (Westoby & Wright, 2003). In our study, differences in LA were paramount in the Myriophyllid, Peplid, Pleustophyte, and Potamid growth‐forms. Apparently, a large‐leaved Potamid or a dense‐leaved Myriophyllid alien can achieve dominance over native species, forming a monolayer or dens canopy and can directly inhibit other species in the competition for light. Interestingly, traits related to growth rate (SLA) were found to be substantial also in the case of these growth‐forms (except Peplids), which suggest that these species produce larger leaves in a faster and easier way.

Former studies have shown that SLA is the most influential trait in the leaf economics spectrum (Saverimuttu & Westoby, 1996), which also reflects relative growth rate (RGR = assimilation rate × leaf mass ratio × specific leaf area). There is also a trade‐off between SLA and leaf life span (leaf longevity) and have a strong relationship with net photosynthesis (i.e., growth rate) (Osnas, Lichstein, Reich, & Pacala, 2013). Therefore, species with high SLA are associated with a strategy where only a small amount of biomass is invested into building short‐lasting structures. Our results suggest that SLA of alien and native aquatic plants differs substantially in almost all growth‐forms (except Nymphaeid and Pepild), which are in line with the results of Lake and Leishman (2004) and Hamilton et al. (2005) who found that high SLA can promote invasiveness. In particular, studies found significant differences even between alien aquatic plant species by their growth rates (Barrat‐Segretain, 2005). The correlation between growth rate and nativeness is contrasting; however, the role of congeners is an important issue in this matter. Further, note that according to Poorter et al. (2009), low SLA value is a general character among aquatic plants, which, based on our results, seems to vary among natives and aliens within growth‐forms. Based on the above, we can presume that the ratio of LA and leaf tissue elements (i.e., LDMC) is limited; water extensively supports leaves of aquatic plants against gravity. This suggestion implies that all leaves have to contain a minimum amount of solid tissue particles even under water to remain compact, and below this limit, species are not able to produce softer leaves to gain more competitive ability. This phenomenon highly expressed in the case of Nymphaeid and Peplid species because these plants exhibited the lowest SLA values in our study. Furthermore, it might represent the case when natives and aliens did not substantially differ within these growth‐forms.

Nitrogen (together with phosphorous) is generally considered to be one of the most limiting elements in terrestrial and aquatic environments. LNC, similar to SLA, also reflects photosynthetic activity (i.e., growth rate) in an alternative way (Cornelissen et al., 2003). In contrast to SLA, LNC represents differences in photosynthetic activity, considering the effectiveness of nutrient recovery. We supposed that alien aquatic plants have an enhanced nitrogen‐use efficiency which was reflected by higher LNC values. Global patterns of leaf N content showed a decline toward the Equator, which indicates a strong relationship with latitude and temperature (Reich & Oleksyn, 2004). Contrary to our hypotheses, our results indicate that nativeness is not related to the photosynthetic activity of aquatic plants in general. However, alien Nymphaeid and Vallisnerid species exhibit higher nitrogen concentrations, which indicate enhanced and more effective photosynthesis and growth rates therein. Moreover, we also justified the latitude and temperature response of leaf N content.

### 4.1 The effect of environmental variables on trait variation and nativeness–traits relationship

The plasticity of certain traits and the diversity of traits are known to depend on the ambient environment (Capers, Selsky, & Bugbee, 2010; Hodgson et al., 2011; Richards, Bossdorf, Muth, Gurevitch, & Pigliucci, 2006). Daehler (2003) found that differences between alien and native species strongly depend on the environment, and the performance of alien species might be better under high resource availability in benign conditions (Richards et al., 2006). Contrary to van Kleunen et al. (2010), who found that the trait relationship of terrestrial species did not depend on the quality of the environment, and the differences between native and alien terrestrial species were robust across environments, our analysis showed the relative importance of abiotic factors in some of the trait–nativeness relationship within some aquatic plant growth‐forms.

The effect of altitude and latitude on aquatic plant diversity and distribution is well known in the literature (e.g., Heegaard, Birks, Gibson, Smith, & Wolfe‐Murphy, 2001; Jones, Li, & Maberly, 2003; Lukács et al., 2015). Temperature and light availability varies on an elevational and latitudinal gradient, and there is a clear trade‐off between altitude and diversity (Jones et al., 2003). Moreover, it has been proven that altitude and latitude (via temperature and precipitation) have an effect on trait variation of terrestrial plant species (Hulshof et al., 2013; Reich et al., 1997). Riis et al. (2012) also pointed out that temperature can affect the competitive ability of the alien *Lagarosiphon major* via phenotypic plasticity. Contrary to that we found, little effect of latitude on the trait variability was found between native and alien aquatic plants, which is presumably due to the short latitudinal gradient. Latitude substantially affected the trait–nativeness relationship only in case of LA within the Pleustophyte growth‐form, but also had a substantial effect on LDMC variation of Myriophyllids.

Aquatic ecosystems at high altitudes are considered as extreme environments in which physical stressors and severe climate may limit the distribution of aquatic plants (Lacoul & Freedman, 2006). However, our study covered a wide elevational gradient (52–1722 m a.s.l.) where we found altitude to be an important factor only in case of LDMC among Myriophyllids and in case of SLA in Potamids. In light of the obtained effect of nativeness, this particularly indicates that nativeness and altitude explain SLA differences together only within Potamids. This is partly in line with the results of Hulshof et al. (2013) who pointed out intra‐ and interspecific variation of SLA along elevational gradients.

The importance of pH has been well documented in aquatic plant ecology; it is related to physiological differences (i.e., the ability to use bicarbonate as carbon source) among species (Madsen & Sand‐Jensen, 1991). The ability of bicarbonate usage and the affinity for bicarbonate vary among species within the same growth‐form and it has a strong intraspecific variability strongly influenced by carbon availability, light, nutrients, and temperature conditions (Hussner, Mettler‐Altmann, Weber, & Sand‐Jensen, 2016; Maberly & Spence, 1983; Sand‐Jensen & Gordon, 1986). We found a trade‐off between pH and LNC (in Vallisnerid), and pH and LDMC (in Myriophyllid), while it also affected the nativeness dependence of LA among Peplid and Pleustophyte species. Considering the proven bicarbonate use ability of many studied Vallisnerid, Myriophyllid, Peplid, and Pleustophyte species, we supposed that the proposed substantial trade‐off between traits and pH might be caused by this attribute of species. It would be in line with those studies that indicate that morphological and physiological attributes of aquatic plants may alleviate the potential carbon limitation of photosynthesis; therefore, many of these species have high LA to unit biomass (i.e., low SLA; Hutchinson, 1975; Nielsen & Sand‐Jensen, 1989).

Previous studies indicated that water depth has a significant influence on individual trait variation (e.g., shoot height, stem dry mass, see Maberly, 1993; Fu et al., 2012), in particular to traits related to the ability of light harvesting and space occupation. Our analyses highlighted the substantial importance of water depth in the relation of nativeness with SLA and LA in case of Pleustophyte and Potamid species, which indicates that the strength of the relationships (aliens tends to have larger leaves and higher growth rate) might decrease with water depth among these species. Our results perfectly fit to the results of Fu et al. (2014) who highlighted that the SLA of aquatic plants is connected to the “niche differentiation” concept (i.e., species use environment differently) along the water depth gradient; and increasing water depth increases the variability of SLA in case of several Potamid (e.g., *Potamogeton pectinatus, P. perfoliatus, and Najas marina*) and Plesutophyte (*Ceratophyllum demersum* and *Hydrocharis dubia*) species.

## 5 Conclusions and Recommendations for Future Research

In this study, we aimed at investigating the functional response of alien aquatic plants, as only a few comparative studies are available which attempted to identify traits governing their success. For instance, Thiébaut (2007) did not find general tendencies in traits for aquatic plants to be more vigorous in their introduced ranges. The seemingly contradicting conclusions of her study could be due to the fact that hydrophyte species of all growth‐forms were pooled together. Generally, alien aquatic plants can be characterized as species which produce larger leaves within shorter time using of fewer nutrients, but these characteristics are not universal throughout all growth‐forms. Also, merging all growth‐forms could hide differences between natives and aliens due to the great diversity between species belonging to different growth‐forms. However, we demonstrated that within certain growth‐forms, alien species have significantly different trait values which enable them to enhance their competitive ability via a more acquisitive plant strategy (i.e., short life cycle and rapid growth rates). Some of the alien aquatic plants invest more in their leaf defense, increasing their structural leaf tissue elements (having larger LDMC). These taxa include alien Herbids such as *Ceratopteris thalictroides*. Other species can increase their growth rate applying a more acquisitive physiology (i.e., lower SLA) like Myriophylloids (e.g., *Myriophyllum aquaticum* and *Cabomba caroliniana*) and Potamids (*Lagarosiphon major, Elodea* spp.), and there are species which can increase their competitive ability by developing larger leaves more quickly like Peplid (e.g., *Rotala rotundifola*), Plesutophyte (*Lemna minuta, Azolla filiculoides*), and Potamid species (*Lagarosiphon major, Elodea* spp.). We also pointed out that environmental variables such as altitude, pH, and water depth are important factors in studied response of nativeness and leaf traits.

The question of identifying traits promoting plant invasiveness is important for understanding plant success in general and also in planning risk assessment protocols and management and preventive actions. We believe that our results provide new insights into what makes an aquatic alien plant to be successful in temperate climate which is expected to inform conservation management strategies or as a base to make an inventory of alien species whose import and placement on the market will be prohibited/permitted (black/white lists). We emphasize that the trait approach we applied here can only partly contribute to the understanding of the mechanism of aquatic plant invasions. Further research is needed to clarify the trait dependence of this issue, especially (i) to explore the phylogenetical dependence of trait differences, (ii) to explore the intraspecific variation of aquatic plant's trait values to obtain finer conclusions, (iii) to collect more functional trait data from aquatic plants to make multitrait comparisons possible, and (iv) to explore the differences in functional community assembly between native and alien aquatic plant communities.

## Conflict of interest

None declared.
